# Non-Covalent Interaction of Folic Acid and 5-Methyltetrahydrofolate with Caseinates Improves the Folates Stability Studied by Multi-Spectroscopic Analysis and Molecular Docking

**DOI:** 10.3390/foods13172756

**Published:** 2024-08-29

**Authors:** Linlin He, Yuqian Yan, Gang Zhang, Yanna Zhao, Fa Zhao, Zhuang Ding, Zhengping Wang

**Affiliations:** 1 Institute of Biopharmaceutical Research, Liaocheng University, Liaocheng 252059, China; helinlin2022@163.com (L.H.); yanyuqian24@163.com (Y.Y.); z19970629g@163.com (G.Z.); ynzhao2011@163.com (Y.Z.); wangzhengping@lcu.edu.cn (Z.W.); 2 Shandong Institute for Food and Drug Control, Jinan 250101, China; zhaofa1987@126.com

**Keywords:** sodium caseinate, folic acid, 5-methyltetrahydrofolate, calcium 5-methyltetrahydrofolate, non-covalent interactions

## Abstract

Folates, a crucial B-group vitamin, serve as a significant functional food supplement. Nevertheless, considerable obstacles persist in improving folates stability in liquid products. In this study, folic acid (FA) and 5-methyltetrahydrofolate (MTFA), two approved sources of folates, were encapsulated with sodium caseinate (NaCas) to enhance their stability. The protective effect of NaCas on folate molecules was investigated using experimental and computational methods. Meanwhile, the influence of divalent calcium ion (Ca^2+^) on the properties of the NaCas-MTFA complex was examined to evaluate the potential application of calcium 5-methyltetrahydrofolate (CaMTFA). Fluorescence tests showed both folates had static quenching behavior and bound to NaCas with a binding constant of 10^4^–10^5^ M^−1^. Hydrophobic interactions were crucial in NaCas-FA complex formation, while hydrogen bonding drove NaCas-MTFA binding. The encapsulation of caseinate notably slowed down the degradation of folates under both light and dark conditions. Moreover, the addition of a low concentration of Ca^2+^ did not adversely impact the binding mechanism of the NaCas-MTFA complex or the degradation curve of MTFA. The results of this study could serve as a valuable resource for the utilization of caseinates in incorporating folates, specifically MTFA, in the creation of natural liquid dietary supplements.

## 1. Introduction

Folate, or vitamin B9, comprises a group of heterocyclic compounds consisting of a pteridine ring, p-aminobenzoic acid, and γ-linked L-glutamic acid, which includes folic acid (FA), tetrahydrofolate, 5-methyltetrahydrofolate (MTFA), and various other derivatives [1]. This essential nutrient is crucial for human metabolism, facilitating normal cell division and growth through processes such as amino acid and nucleotide synthesis, as well as one-carbon unit metabolism [2]. Due to its source facilitation and notable chemical stability, FA, a synthetic oxidized form of folate, is presently the most commonly utilized form of folate in fortification food and pharmaceutical products [1]. In recent years, MTFA has garnered increased attention from researchers, consumers, and food manufacturers. MTFA, as a primary natural form of folate, offers several advantages such as direct utilization without the need for metabolism [3], prevention of masking of vitamin B12 deficiency [4], and mitigation of cardiovascular risks in perinatal women [5]. However, both FA and MTFA are susceptible to degradation in aqueous environments when exposed to oxygen, light, pH variations, and temperature fluctuations. This degradation is primarily attributed to photo-induced cleavage of the C9–N10 bond and oxygen-induced decomposition of the pteridine ring [1]. Consequently, these factors present significant challenges for the preservation of FA and MTFA during food processing and storage.

The utilization of protein-based complexes as carriers is a promising strategy for safeguarding bioactive compounds from degradation and enhancing their bioavailability [6]. Through strong non-covalent interactions, macromolecular proteins effectively capture and encapsulate small molecules, thereby creating a protective barrier against degradation and enhancing their dispersibility and solubility [7,8]. Caseins, comprising approximately 80% of milk protein, are considered versatile and environmentally friendly biopolymers. Sodium caseinate (NaCas) is a commonly utilized casein product in the food industry, obtained through the acid-precipitation of casein followed by neutralization with sodium hydroxide for spray drying [9]. Owing to its high water dispersibility, cost-effectiveness, safety profile, and nutritional benefits, NaCas is widely used in liquid food production. Therefore, NaCas has been extensively studied as a protein carrier for encapsulating various bioactive components, such as poorly soluble carotenoids, curcumin, docosahexaenoic acid, vitamin D, and soluble polyphenolic compounds [10,11]. Rashidinejad et al. [12] demonstrated that the incorporation of NaCas significantly improved the solubility and chemical stability of rutin. Similarly, Ghayour et al. [13] observed that the addition of caseinates significantly enhanced the chemical stability of phenolic compounds during an accelerated shelf-life test. These findings suggest that caseinates can function as effective protective carriers for the encapsulation and delivery of labile bioactive compounds. Several studies have addressed the NaCas-FA non-covalent complexes. Penalva et al. [14] reported the formation of NaCas-FA non-covalent nanoparticles, which demonstrated the ability of the NaCas-based carrier to provide a gastro-resistant barrier for FA and achieve targeted intestinal release. Malekhosseini et al. [15] showed a high affinity between NaCas and FA, with a binding constant of 10^5^ M^−1^ at pH 7.4. Previous research has been conducted on the interaction between natural caseins and FA. Bourassa and Tajmir-Riahi [16] determined binding constants of 4.8 × 10^4^ and 7.0 × 10^4^ M^−1^ for the interaction of FA with α-caseins and β-caseins, respectively. Zhang et al. [17] observed that the binding of FA to β-casein effectively hindered the photo-decomposition of FA under UV-light exposure. To the best of our knowledge, no studies have yet been conducted on the protein-MTFA complex and the protective effect of protein on MTFA. It is hypothesized that FA and MTFA bind to caseinates through non-covalent interactions with high binding affinity, thereby effectively mitigating folate degradation.

The metal divalent cations, specifically calcium (Ca^2+^) and magnesium (Mg^2+^) ions, exhibit a high affinity for casein, leading to significant effects on the aggregation state and size of casein particles through mechanisms such as cross-linking, micellization, and flocculation. These interactions can have a profound impact on the binding of proteins and small molecules [18,19]. Dickinson and Casanova [20] observed the reversible self-association of caseinate, which is influenced by variations in temperature and calcium levels. Li et al. [18] also noted a similar flocculation of β-casein when exposed to Ca^2+^ and heat, resulting in a decrease in the emulsifying ability of β-casein. The commercially available form of MTFA is predominantly administered as calcium 5-methyltetrahydrofolate (CaMTFA). CaMTFA, utilized as a food additive, introduces Ca^2+^ into protein-based food matrices, potentially leading to alterations in protein structure and polymerization, consequently impacting the interactions and complexes formed between proteins and small molecules. However, there is a lack of research on the influence of Ca^2+^ on NaCas-folate complexes.

Hence, the aim of this study is to investigate the binding mechanism between NaCas and two important folate molecules (FA and MTFA). Their interactions were studied using multi-spectroscopic techniques and molecular docking to better elucidate the effect on the chemical stability of folate. Furthermore, the influence of Ca^2+^ incorporation on the interaction of NaCas-folate complexes was investigated. To our knowledge, this is the first report on the interaction and properties of NaCas-MTFA complexes with and without Ca^2+^. The insights gained from this research contribute to the current understanding of folate behavior when interacting with caseinate and inform formulation strategies for commercially available folate-fortified beverages.

## 2. Materials and Methods

### 2.1. Materials

Folic acid (FA, purity ≥97%; CAS# 59-30-3; MW 441.4) was purchased from Macklin (Shanghai, China). 5-Methyltetrahydrofolate (MTFA, purity ≥98%; CAS# 31690-09-2; MW 459.47) and calcium 5-methyltetrahydrofolate (CaMTFA, purity ≥95%; CAS# 151533-22-1; MW 497.52) were purchased from Aladdin (Shanghai, China). Sodium caseinate (NaCas, CAS# 9005-46-3; Lot# C8654) was purchased from Sigma-Aldrich (St. Louis, MO, USA). 8-Anilino-1-naphthalenesulfonic acid (ANS, ≥96%) was obtained from Macklin (Shanghai, China). High-performance liquid chromatography (HPLC)-grade methanol was purchased from Fisher Scientific (Pittsburgh, PA, USA). All other chemicals used in this study were of analytical grade and were purchased from Macklin (Shanghai, China). The water used in this study was purified by a Millipore Milli-Q system (Bedford, MA, USA).

### 2.2. Sample Preparation

The NaCas powder was dissolved in 10 mM phosphate buffer (pH 7.4), stirred at room temperature for two hours, and then placed at 4 °C overnight to ensure complete dissolution and full molecular hydration, resulting in a 20 μM NaCas stock solution. The apparent molecular mass of NaCas is reported to be an average molecular weight of 23.6 kDa [21]. Three types of folate powders were separately dissolved in 10 mM phosphate buffer (pH 7.4), stirred at room temperature for 30 min until fully dissolved, to obtain 50 μM folate stock solutions, which were freshly prepared before each experiment. The protein and folate stock solutions were mixed in varying proportions and subsequently diluted with 10 mM phosphate buffer (pH 7.4) to achieve the final concentrations required for subsequent experiments. The mixtures were vortexed for 20 s to facilitate the formation of protein-folate complexes. All samples were prepared in plastic tubes, incubated for approximately 30 min at room temperature, and covered with aluminum foil prior to analysis. All NaCas-folate complexes were independently prepared three times for subsequent analysis.

### 2.3. Fluorescence Spectroscopy

Various volumes of FA/MTFA stock solutions (50 μM) were mixed with the fully hydrated NaCas stock solution (20 μM) and further diluted using a 10 mM phosphate buffer (pH 7.4) to formulate NaCas-FA/MTFA complex solutions. The final concentration of NaCas in these solutions was standardized to 10 μM, while the final concentrations of FA/MTFA varied between 0 and 25 μM. The resulting molar ratios of FA/MTFA to NaCas were 1:2, 1:1, 1.5:1, 2:1, and 2.5:1. To simulate the application scenario of CaMTFA, CaCl_2_ was added as another solute to the NaCas-MTFA system for determining the influence of Ca^2+^ on the complexes. To prevent interference caused by changes in Ca^2+^ concentration, its concentration was maintained at 25 μM by adding CaCl_2_ instead of CaMTFA powder. The 25 μM Ca^2+^ concentration was considered equivalent to the maximum concentration of MTFA used in fluorescence analysis. A stock solution of CaCl_2_ (500 μM) was prepared by dissolving the powder in a 10 mM phosphate buffer (pH 7.4). Subsequently, 0.15 mL of this stock solution was introduced into the MTFA and NaCas-MTFA systems to form the simulated CaMTFA and NaCas-CaMTFA complexes, respectively.

The fluorescence spectra were acquired utilizing an F-7000 spectrofluorometer (Hitachi, Tokyo, Japan), which was equipped with a xenon lamp source, a 10 mm quartz cuvette, and a thermostatic water bath. The samples underwent a 30-min incubation period in the thermostatic water bath at three distinct temperatures: 298.2 K, 303.2 K, and 308.2 K, prior to measurement. The samples were excited at 280 nm, and the emission spectra were recorded in the range of 300–500 nm with a slit width of 5 nm. The fluorescence spectra were acquired at a voltage of 490 V with a scan rate of 1200 nm min^−1^. Initially, the emission spectrum of NaCas (10 μM) was recorded in the absence of any ligands. To elucidate the binding characteristics, subsequent emission spectra of NaCas were measured in the presence of ligands at concentrations ranging from 5 to 25 μM. Each trial was performed in triplicate. Fluorescence intensity was normalized relative to that of 10 μM NaCas at the emission maximum. The fluorescence spectrum of the solvent, measured under identical conditions, served as a blank for background correction of the sample fluorescence spectra. To offset the inner-filter effect, the absorbance of samples was measured using a UV-visible spectrometer (U-3900H, Hitachi, Tokyo, Japan). The fluorescence intensities were corrected using the following Equation (1) [22], where F_obs_ and F_corr_ are the fluorescence intensities before and after correction, respectively. A_em_ and A_ex_ are the absorbance at the emission and excitation wavelengths, respectively.

F_corr_ = F_obs_ × e^(Aem+Aex)/2^ (1)

### 2.4. Surface Hydrophobicity

The surface hydrophobicity (S_0_) of NaCas with and without two folate molecules was determined using the 1-anilino-8-naphthalenesulfonic acid (ANS) fluorescence probe method. Sample solutions containing NaCas (0–30 µM) and corresponding complexes containing 25 µM folate molecules (FA, MTFA, and CaMTFA) were prepared and incubated for 30 min at 25 °C. Subsequently, 4 mL of each sample was mixed with 40 μL of ANS solution (8 mM) and incubated in the dark for 30 min at 25 °C. The fluorescence intensity of the samples was measured within the emission range of 485 nm using an F-7000 spectrofluorometer at an excitation wavelength of 390 nm. Each sample’s fluorescence intensity was corrected by subtracting the fluorescence intensity of the corresponding sample without the ANS probe. Each trial was performed in triplicate. The slope of the curve of the fluorescence intensity versus protein concentration was calculated through linear regression analysis (R^2^ > 0.99) and utilized as the protein S_0_ value [23].

### 2.5. Fourier Transform Infrared (FTIR) Spectroscopy

The FTIR spectra (400–4000 cm^−1^) of the folate-NaCas complexes and NaCas sample were obtained using a Nicolet iS50 FTIR spectrometer (Thermo Fisher Scientific, San Jose, CA, USA). The folate-NaCas complexes, with a concentration of 25 μM for each component, were prepared and incubated at 25 °C for 30 min. Subsequently, the complex samples were frozen in liquid nitrogen and dried in a VirTis vacuum freeze-dryer (SP Industries, Warminster, PA, USA). The dried sample (1.0 mg) was mixed with KBr powder (100 mg), ground together, and pressed into a flake for FTIR analysis. The secondary structure of the protein was calculated based on the secondary derivative in the 1600–1700 cm^−1^ amide I band region using PeakFit software version 4.12 (Thermo Fisher Scientific).

### 2.6. Molecular Docking

Given that αs1-casein and β-casein constitute over 70% of the total casein content, they were selected as representative molecules for conducting molecular docking studies between caseinate and FA/MTFA. Due to the unavailability of crystal structures for αs1-casein and β-casein, these structures were generated using the I-TASSER server (https://zhanglab.ccmb.med.umich.edu/I-TASSER) (accessed on 15 April 2024). The amino acid sequences of αs1-casein (GenBank: ACG63494.1) and β-casein (GenBank: AAA30431.1) were retrieved from the National Center for Biotechnology Information (https://www.ncbi.nlm.nih.gov/protein) (accessed on 15 April 2024) [24]. The PROCHECK program was employed to validate and assess the quality of the three-dimensional protein models (https://saves.mbi.ucla.edu) (accessed on 15 April 2024). The initial three-dimensional configuration of FA/MTFA (Pubchem CID: 135398658/135398561) was retrieved from PubChem Compound (https://pubchem.ncbi.nlm.nih.gov) (accessed on 15 April 2024). The structures of αs1-casein and β-casein were optimized and extended to three-dimensional structures using Open Babel. The protonation status of all compounds was established at pH = 7.4. The chemical structures of FA and MTFA, serving as ligands, were imported into the AutoDock Tool (ADT3). Subsequent to the adjustment of charges, identification of flexible torsions, and determination of the ligand root, the structures were saved in pdbqt format for ensuing molecular docking studies. Similarly, αs1-casein and β-casein were selected as receptors, and their structures were imported into ADT3. Following the calculation of charges and addition of atom types, the receptor structures were also saved in pdbqt format for molecular docking analysis. The docking box was generated using the AutoGrid (version 8.9). Subsequently, molecular docking was performed utilizing the default parameters of AutoDock Vina version 1.2.0 (Scripps Research, La Jolla, CA, USA). The conformation with the lowest calculated binding free energy was selected as the optimal binding conformation, and the interactions were analyzed. The scores of all complex conformations resulting from the molecular docking are presented in Table S1. Finally, the protein-ligand interaction diagram was generated using PyMOL v3.0.4 (Schrödinger, Inc., New York, NY, USA). The proteins are represented as dark blue cartoon models, while the ligands are depicted as cyan stick models, and their binding sites are illustrated as magenta stick structures. Hydrogen bonds, ionic interactions, and hydrophobic interactions are indicated by yellow, magenta, and green dashed lines, respectively.

### 2.7. Storage Stability

#### 2.7.1. Sample Storage

The chemical stability of FA and MTFA, as well as the physical stability of the NaCas-folate complexes, were investigated using an MKF720 climate chamber (Binder GmbH, Tuttlingen, Germany) at 25 °C with and without light exposure. The concentration of each component in the complexes was maintained at 10 μM. Solutions containing only folate molecules were used as the blank groups. All samples were supplemented with sodium azide (0.01 wt.%) to prevent microbial growth. In the presence of light, a 5 mL sample containing either FA or MTFA was placed in a 10 mL sealed glass vial and exposed to built-in incandescent light (30 cm distance, 200 lux) for 7 days (FA) and 3 days (MTFA). Under conditions of light exclusion, the stability test duration was 7 weeks (FA) and 7 days (MTFA) respectively.

#### 2.7.2. Physical Stability

The physical stability of the complex sample was assessed by measuring the average particle size and ζ-potential of the complexes before and after storage using a Zetasizer Nano system (Malvern Instruments, Malvern, UK), with analysis conducted without dilution or filtration. The Z-average particle size was determined using dynamic light scattering and cumulant analysis in Zetasizer software version 7.04.

#### 2.7.3. Chemical Stability

During the storage period, 1 mL aliquots of the sample were collected to analyze the remaining content of FA and MTFA. Analytical curves for FA and MTFA content (1–500 μM, R^2^ > 0.9997) were generated using a Dionex Ultimate 3000 HPLC system (Thermo Fisher Scientific) with a C18 analytical column (QuikSep SP ODS-AQ, 5 μm, 4.6 mm × 250 mm, H&E Technology, Beijing, China). The mobile phase was composed of a mixture of methanol and a 0.1% phosphoric acid solution, with a ratio of 30/70 (*v*/*v*) for FA and 20/80 (*v*/*v*) for MTFA. The flow rate and detection wavelength were set at 1.0 mL/min and 280 nm, respectively. The retention rate (RS) of FA and MTFA was determined using the following Formula (2) [25], where C_s_ is the remaining amount of folate molecules (μM), and C_0_ is the initial amount of folate molecules (10 μM).

RS (%) = C_s_/C_0_ (2)

### 2.8. Differencial Scanning Calorimetry (DSC)

Thermal analysis was conducted using Q2000 DSC system (TA Instruments, New Castle, DE, USA) under a nitrogen atmosphere. The corresponding dried powder of the NaCas-folate complexes were prepared according to Section 2.5. The tested powder (3–5 mg) was placed within a covered but not tightly sealed aluminum pan. DSC runs were performed from 25 to 300 °C at a rate of 10 °C/min with a 50 mL/min nitrogen purge. The DSC system was calibrated using an empty aluminum pan as a reference. The resulting DSC curves were analyzed using Universal Analysis software (TA Instruments).

### 2.9. Statistical Analysis

The data were presented as mean ± standard deviation (SD, n = 3) and analyzed using one-way analysis of variance (ANOVA) with SPSS software version 19.0. Significant differences were determined using a *t*-test, with results considered statistically significant at a threshold of *p* < 0.05.

## 3. Results and Discussion

### 3.1. Quenching Mechanism of NaCas by Folate

**Figure 1:**
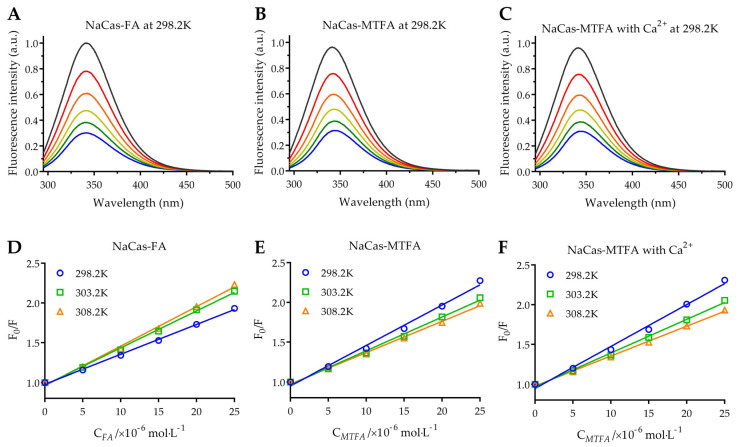
(**A**–**C**) Normalized fluorescence quenching spectra of 10 μM sodium caseinate (NaCas) with 0–25 µM folic acid (FA) (**A**) and 5-methyltetrahydrofolate (MTFA) (**B**), as well as MTFA under 25 µM divalent calcium ion (Ca^2+^) (**C**) at 298.2 K. (**D**–**F**) Stern-Volmer plots for the NaCas-folate system at 298.2, 303.2, and 308.2 K.

The intrinsic fluorescence of NaCas is primarily attributed to tryptophan (Trp) residues, including Trp164 and Trp199 in αs1-casein, Trp109 and Trp193 in αs2-casein, Trp143 in β-casein, and Trp76 in κ-casein [13]. The binding of small molecules with proteins leads to the quenching of tryptophan fluorescence, making it a common method for studying protein-small molecule interactions. This study investigates the interaction between NaCas and three types of folates using fluorescence spectroscopy of NaCas under conditions of pH 7.4 and temperatures of 298.2, 303.2, and 308.2 K. The fluorescence spectra of NaCas were analyzed at various concentrations of folate (folate/protein molar ratios ranging from 0 to 2.5) as shown in Figure 1A–C. It was observed that the fluorescence intensity of NaCas decreased gradually with increasing concentrations of folate molecules. In the presence of 25 μM folate molecules, the fluorescence intensities of NaCas (10 μM) decreased by 67.3–69.8%, indicating a strong binding affinity between folate molecules and NaCas. Moreover, the interaction between NaCas and folate molecules resulted in a slightly bathochromic shift in the maximum emission wavelength of NaCas, suggesting that the presence of folates leads to an increase in the hydrophilicity of the micro-environment surrounding Trp residues [26,27].

The fluorescence quenching mechanism can be classified into two categories: static quenching, which occurs due to the formation of a ground-state complex between the fluorophore and the quencher, and dynamic quenching, which is dependent on their diffusion and collision interactions [28]. The mechanism of fluorescence quenching can be elucidated using the Stern-Volmer Equation (3), in which F_0_ and F represent the corrected fluorescence intensities of protein and protein-ligand complexes, respectively. The equation includes parameters such as *K*_sv_, the Stern-Volmer quenching constant (L·mol^−1^); *K*_q_, the quenching rate constant (L·mol^−1^·s^−1^); [Q], the molar concentration of folate (mol·L^−1^); and τ_0_, the average lifetime of the biomolecule complexes in the absence of any quencher (τ_0_ = 10^−8^ s) [29].

F_0_/F = 1 + *K*_SV_·[Q] = 1 + *K*_q_·τ_0_·[Q] (3)

The Stern-Volmer plots of the NaCas-folate systems demonstrate a strong linear relationship (Figure 1D–F, R^2^ > 0.98), indicating a single quenching mechanism of folate to NaCas [30]. The quenching constant *K*_q_ values in the NaCas-folate systems ranged from 3.76 to 5.27 × 10^12^ L·mol^−1^·s^−1^, significantly exceeding the maximum collision quenching constant of biomolecules (2 × 10^10^ L·mol^−1^·s^−1^). These results suggest that the fluorescence quenching of NaCas by folate molecules is predominantly static, characterized by the formation of biomolecule complexes with high binding affinity [31]. This is supported by the research conducted by Chilom et al. [32], who utilized fluorescence spectroscopy to investigate the interaction between FA and bovine serum albumin (BSA) and confirmed static quenching in the BSA-FA complex with with the *K*_q_ value of 13.2 × 10^12^ L·mol^−1^·s^−1^.

### 3.2. Thermodynamic Parameters of the NaCas-Folate System

The Lineweaver-Burk Equation (4) can be utilized to determine the binding constant (*K*_a_) and number of binding sites (n) in a static quenching mechanism of a bimolecular complex [33]. This equation involves the fluorescence intensities (F_0_ and F) in the absence and presence of folates, respectively. The free concentration of folates ([Q]) can be calculated using Equation (5), which considers the total concentrations of folate molecules ([Q]_0_) and NaCas ([P]_0_) [34].

log(F_0_/F − 1) = log*K*_a_ + n·log[Q] (4)

[Q] = [Q]_0_ − n·[(F_0_−F)/F_0_]·[P]_0_ (5)

As indicated in Table 1, the binding constants of folate molecules to NaCas ranged from 7.48 to 13.70 × 10^4^ M^−1^, consistent with the findings of Bourassa et al. [35], who investigated the binding interaction of FA with α- and β-caseins and reported *K*_a_ values of 4.8 × 10^4^ M^−1^ and 7.0 × 10^4^ M^−1^ for α-caseins-FA and β-caseins-FA, respectively. At 298.2 K, the NaCas-MTFA complexes exhibited a higher *K*_a_ compared to NaCas-FA, suggesting that MTFA displayed a stronger binding affinity with NaCas at room temperature. The reduced pteridine ring in MTFA exhibits enhanced structural flexibility, containing an additional imino group and N-methyl moiety. These structural factors likely facilitate a tighter binding interaction between MTFA and proteins, potentially forming additional hydrogen bonds with specific amino acid residues. Furthermore, the observed n values approaching 1.0 in bimolecular complexes indicate a 1:1 ration of caseinate-folate molecules binding.

Based on the *K*_a_ values of NaCas-folate complexes at three temperatures, the thermodynamic parameters of their interaction, including Gibbs free energy change (ΔG), enthalpy change (ΔH) and entropy change (ΔS), were calculated by Van’t Hoff (6) and the Gibbs-Helmholtz Equation (7) [36]. The experimental temperature in Kelvin (T) and the gas constant (R = 8.314 J·mol^−1^·K^−1^) were utilized in these calculations.

ln*K*_a_ = − ΔH/(R·T) + ΔS/R (6)

ΔG = ΔH − T·ΔS = − R·T·ln*K*_a_ (7)

The linear van’t Hoff plots illustrating the bimolecular interaction were presented in Figure S1, with corresponding thermodynamic parameters detailed in Table 1. As the testing temperature increased, the *K*_a_ values of NaCas-FA complexes exhibited a gradual increase, while the *K*_a_ values of NaCas-MTFA complexes showed a gradual decline, suggesting a difference in the binding mechanisms of FA and MTFA with NaCas. First, the ΔG of all complexes are negative across various conditions, indicating the spontaneous binding action of folates on NaCas.

The values of ∆H and ∆S were determined to be 21.65 ± 0.49 kJ·mol^−1^ and 166.30 ± 1.62 kJ·mol^−1^ for the binding of NaCas-FA, and −45.62 ± 1.30 kJ·mol^−1^ and −54.79 ± 4.29 kJ·mol^−1^ for the interaction of NaCas-MTFA. These results suggest that hydrophobic interactions may be the primary driving forces in the NaCas-FA binding, while hydrogen bonds and van der Waals forces are significant contributors in the NaCas-MTFA complex [37]. The molecular structure of folates contains both hydrophobic aromatic rings and various polar hydrophilic groups, such as amino, hydroxyl, and carboxyl groups. The interaction mechanisms between small-molecular ligands containing various active groups and proteins can exhibit a range of diverse binding modes [33,38]. To elucidate the primary binding mechanism of folates and NaCas, protein surface hydrophobicity analysis and molecular docking simulations were subsequently conducted.

Additionally, the NaCas-MTFA complex with Ca^2+^ displayed similar *K*_a_ values and thermodynamic parameters to the NaCas-MTFA complex, suggesting that the presence of Ca^2+^ (25 μM) did not significantly impact the binding affinity between MTFA and NaCas. The presence of divalent cations (e.g., Ca^2+^ and Mg^2+^) influences the conformation and aggregation of casein molecules through cross-linking and charge-neutralizing effects, indirectly affecting their ability to bind ligands [39,40]. Although different casein molecules exhibit varying sensitivity to Ca^2+^, a relatively high Ca^2+^ concentration is required to induce changes in the physicochemical properties of casein molecules (3–8 mM for αs1-casein; 2 mM for αs2-casein, and higher levels for β-casein) [41]. Considering the recommended intake (~0.9 μmol/day for adults) of folates as a nutritional supplement, the concentration of folates in liquid food is generally low. In our study, the binding behavior of MTFA to NaCas was examined in a liquid environment with 25 μM Ca^2+^ to facilitate the application of CaMTFA as a folate source. The results showed that a low concentration of Ca^2+^ did not significantly affect the MTFA-NaCas interaction.

### 3.3. Surface Hydrophobicity

**Figure 2:**
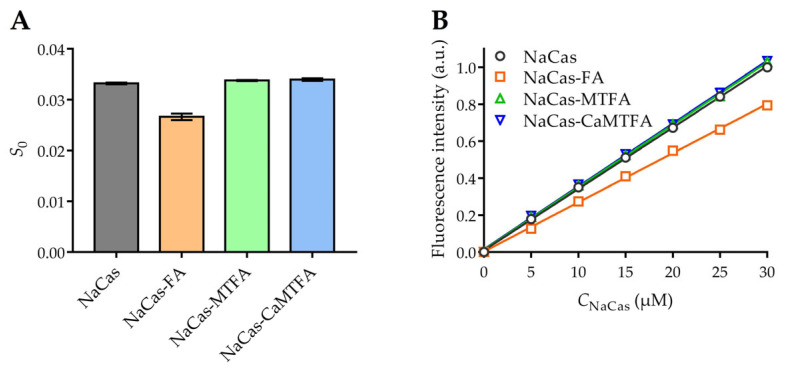
(**A**) Surface hydrophobicity (S_0_) of NaCas alone and NaCas-folate complexes. (**B**) Variation in relative fluorescence intensity of 1-anilino-8-naphthalenesulfonic acid (ANS) probe with 0–30 µM concentrations of NaCas alone and NaCas-folate complexes. Each trial was repeated in triplicate. The fitting results of each trial are shown in Table S3.

The hydrophobicity of the protein surface (S_0_) depends on the presence of non-polar amino acid residues that are exposed on the protein surface, potentially serving as binding sites for small molecules through hydrophobic interactions [42]. The hydrophobic surface characteristics of both NaCas and NaCas-folate complexes were assessed using the hydrophobic probe bis-ANS (Figure 2). In comparison to NaCas alone, the addition of FA resulted in a 20.6% reduction in protein S_0_, which was ascribed to the interaction between NaCas and FA. This interaction involved the occupation of hydrophobic regions on the protein surface by FA, consequently diminishing the quantity of binding sites accessible for ANS [43]. This reduction in protein S_0_ was commonly observed in complexes formed between proteins and hydrophobic molecules [44]. The inclusion of MTFA and CaMTFA did not significantly impact the surface hydrophobicity of NaCas (*p* > 0.05), suggesting that the ANS-accessible hydrophobic regions on the surface of NaCas were not masked by MTFA. This observation implies that hydrophobic interactions may not be the predominant driving force in the interaction between NaCas and MTFA, a conclusion supported by the findings of fluorescence analysis.

### 3.4. Fourier Transform Infrared (FTIR) Spectra

The FTIR spectra of NaCas-folate complexes were analyzed in the 4000–400 cm^−1^ region and presented in Figure S2. Comparison with pure NaCas revealed no new absorption peaks in the spectra of NaCas-folate complexes, suggesting that the inclusion of folates did not result in the formation of covalent bonds. The broad peak observed at approximately 3400 cm^−1^ in the NaCas spectra is attributed to the stretching vibrations and intermolecular hydrogen bonding of O–H and N–H [45]. The characteristic peak observed in the NaCas-MTFA and NaCas-CaMTFA complexes was shifted to 3377.83 and 3377.78 cm^−1^, respectively, suggesting the involvement of hydrogen bonds in the interaction between MTFA and NaCas [46,47]. Conversely, a similar shift was not observed in the NaCas-FA complex.

**Figure 3:**
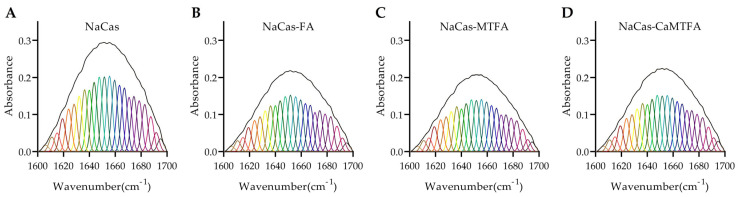
Amide I band (1600–1700 cm^−1^) and the fitted band components of sodium caseinate (NaCas) alone (**A**) and NaCas complexes with folic acid (FA), 5-methyltetrahydrofolate (MTFA), and calcium 5-methyltetrahydrofolate (CaMTFA) (**B**–**D**).

The amide I band region (1600–1700 cm^−1^) in the FTIR spectrum is commonly utilized for analyzing the secondary structure of proteins. The integral areas of the four component bands within the amide I region were calculated to correspond with the classic protein secondary structures: β-sheet (1600–1640 cm^−1^), random coil (1640–1650 cm^−1^), α-helix (1650–1660 cm^−1^), and β-turn (1660–1700 cm^−1^) [48]. The analysis of the amide I band regions of NaCas-folate complexes, as depicted in Figure 3, revealed the calculated protein secondary structures summarized in Table 2. The percentages of α-helix, β-sheet, β-turn, and random coil in NaCas were found to be 19.60, 25.71, 36.06, and 18.63%, respectively, indicating a higher β-turn content. The presence of folates did not result in significant changes to the secondary structure of NaCas. Caseins, being proline-rich proteins with limited secondary and tertiary structures, exhibit a certain degree of conformational stability and resistance to denaturation [49]. Yi et al. found that the interaction with lutein had minimal effect on the secondary structures of NaCas, despite a binding constant of 10^5^ M^−1^ [50].

### 3.5. Molecular Docking Study

Molecular docking serves as a valuable tool in elucidating the binding sites and strengths of small molecules on large biomolecules. In this study, three-dimensional models of αs1-casein and β-casein were initially constructed and validated using the PROCHECK program. Analysis of the Ramachandran plot revealed that 95.7% and 94.0% of amino acids in αs1-casein and β-casein, respectively, were situated within optimal and allowed regions, indicating the reliability of the predicted structural framework for these proteins (Figure S3) [51]. MTFA and FA, utilized as small-molecular ligands in molecular docking, were selected because CaMTFA exists in solution as MTFA and calcium ions. The calculated lowest binding free energies for FA docking with αs1-casein and β-casein were −28.9 kJ·mol^−1^ and −35.6 kJ·mol^−1^, respectively, while for MTFA they were −30.1 kJ·mol^−1^ and −37.2 kJ·mol^−1^, respectively. These values are consistent with the Gibbs free energy changes obtained in fluorescence analysis, indicating that the protein-ligand interactions occur spontaneously.

**Figure 4:**
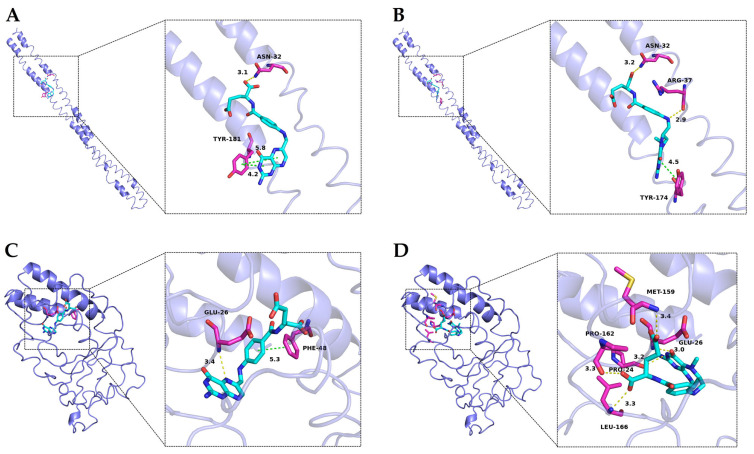
Molecular docking conformation of folic acid (FA) and 5-methyltetrahydrofolate (MTFA) with α_s1_-casein (**A**,**B**) and β-casein (**C**,**D**). The left and right images show the high-affinity site and detailed view of the surrounding amino acids of folates.

The localization of folate docking sites on casein is illustrated in Figure 4. Both FA and MTFA exhibited a similar binding site on αs1-casein, although they displayed distinct binding conformations with the amino acids within the binding cavity. Specifically, the pteridine ring of FA formed a stronger hydrophobic interaction with Tyr-181 of αs1-casein, while its glutamic acid group engaged in a hydrogen bond with Asn-32. In contrast, MTFA interacted with Asn-32 and Arg-37 through the formation of multiple hydrogen bonds. The binding interactions between FA and MTFA with β-casein exhibited notable differences. FA exhibited hydrophobic bonding between its middle benzene ring and Phe-48, as well as a hydrogen bond interaction between the 5′-tertiary amine in the pteridine ring. In contrast, MTFA formed five hydrogen bonds with pro-24, glu-26, Met-159, Pro-162, and Leu-166 of β-casein through its carbonyl and oxhydryl groups, with no significant hydrophobic interactions observed. The findings from molecular docking indicate a greater presence of hydrophobic interactions in the formation of NaCas-FA complexes, while hydrogen bonding interactions predominantly drive the binding between NaCas and MTFA. This observation aligns with the results obtained from fluorescence analysis, indicating that non-covalent interactions between caseinates and folates play a crucial role in the formation of complexes. Hydrophobic interactions and hydrogen bonding have been identified as the primary driving forces governing the non-covalent interactions. In protein-ligand complexes, hydrophobic interactions predominantly occur between the aliphatic and aromatic amino acids of the protein and the benzene rings and hydrophobic groups of small molecules. In contrast, hydrogen bonding mainly forms among the carbonyl (C=O), hydroxyl (-OH), and amino (-NH_2_) groups of the protein’s peptide chain and the corresponding groups of small molecules [52]. These interactions not only stabilize the active groups of small molecules, thereby enhancing the chemical stability of labile molecules, but also improve the functional properties of proteins, such as anti-degradation and emulsifying activity [53].

### 3.6. Physical Stability of Complexes

The particle sizes and ζ-potential of the complex systems were analyzed to evaluate their physical stability (Table 3). The initial particle size of NaCas was determined to be 168.6 ± 7.0 nm, a value consistent with the findings of Wang et al. [54] who reported a size of 168.7 ± 3.0 nm. Previous research by Mantovani et al. [21] indicated that the hydrodynamic diameter of caseinate monomers is approximately 20 nm; nevertheless, caseinate molecules in aqueous solutions have the ability to self-assemble into micelles-like aggregates ranging from 100 to 200 nm in size, even at very low concentrations of 0.1 mg mL^−1^ [55]. In this study, the concentration of caseinate in the solutions was set at 10 μM (equivalent to approximately 0.24 mg mL^−1^), indicating the potential formation of nanoparticle aggregates composed of caseinate particles. Following the addition of folate molecules at a concentration of 10 µM, the sizes of the complex systems exhibited a minor increase from 165.6 to 174.6 nm, with no significant alterations observed in the ζ-potential values.

**Figure 5:**
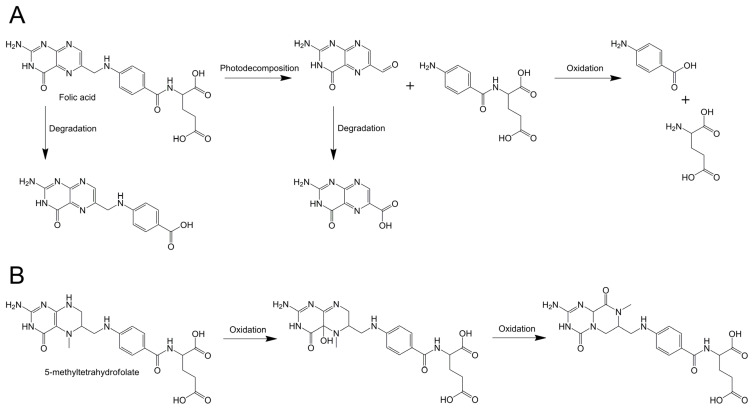
The proposed degradation pathway of folic acid (FA) (**A**) and 5-methyltetrahydrofolate (MTFA) (**B**) during storage.

After a period of storage, the particle sizes of the NaCas-folate complex system exhibited varying degrees of decrease (137.4–144.9 nm), accompanied by a significant increase in the absolute values of their negative ζ-potential. This phenomenon may be attributed to alterations in the protein particles’ surrounding environment induced by newly formed folate-associated derivatives. Throughout the storage duration, FA and MTFA molecules underwent photo-decomposition and oxidation, resulting in the generation of diverse degradation products (Figure 5) [1,56]. In the study conducted by Liang et al. [57], it was discovered that the complexes of FA and three milk proteins exhibit binding not only with FA but also with its degradation products, leading to alterations in protein structure and degradation. The increase in ζ-potential absolute value provides a stronger electrostatic repulsion among charged particles, promoting particle dispersion with lower aggregation [58]. This could be another reason for the decline of the particle size in the complexes during storage.

### 3.7. Chemical Stability of Folates

**Figure 6:**
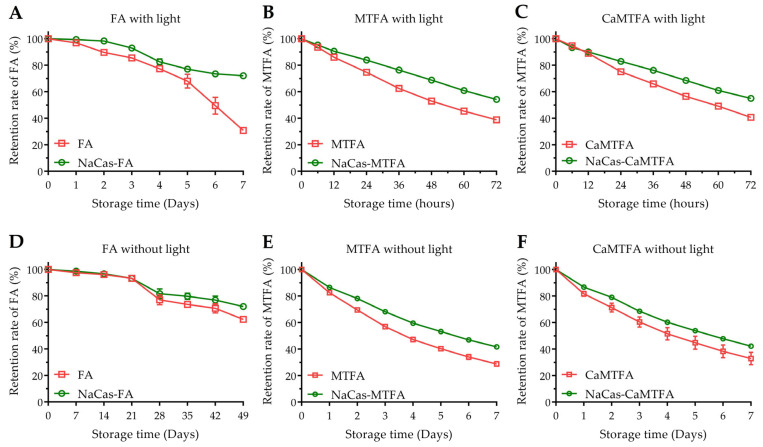
Chemical stability of folic acid (FA) (**A**,**D**), 5-methyltetrahydrofolate (MTFA) (**B**,**E**), and calcium 5-methyltetrahydrofolate (CaMTFA) (**C**,**F**) in sodium caseinate (NaCas)-folate complexes during storage at 25 °C with and without light conditions.

Folates are susceptible to degradation by oxygen, heat, and light, leading to the formation of inactive non-pteridinic products, thereby significantly restricting their utility in the food industry. The retention rates of various folate types (FA/MTFA/CaMTFA) were assessed in the presence and absence of NaCas under both light and dark conditions (Figure 6). After seven weeks of storage with protection from light, the remaining FA content in the blank group decreased to 62.3 ± 0.4% of the initial amount, while 72.0 ± 1.7% of FA in NaCas-FA complexes was retained.

Exposure to light increases the susceptibility of FA to photo-decomposition through C_9_-N_10_ bond cleavage between the pteridine ring and p-aminobenzoic acid [59]. After 7 days of light exposure, the FA content in the blank group decreased rapidly to 30.9 ± 0.5%, while that in the complexes increased to 72.1 ± 0.7%. Due to the reduced pteridine ring in its structure, MTFA is more easily oxidized, resulting in lower chemical stability compared to FA. When shielded from light, the MTFA content decreased to 56.8 ± 1.3% after 3 days and 28.7 ± 1.7% after 7 days. The degradation of MTFA was accelerated under light conditions, resulting in a decrease in MTFA concentration to 38.9 ± 0.1% after 3 days (72 h). Conversely, the presence of NaCas improved the retention rate of MTFA under both dark (41.7 ± 0.7% after 7 days) and light conditions (54.2 ± 0.2% after 3 days). These results indicate that the incorporation of NaCas effectively inhibits the decomposition of folates, leading to a reduction in the degradation rate of folates (*p* < 0.05). Liang et al. [57] and Zhang et al. [17] documented the protective properties of some milk protein components, such as β-lactoglobulin, α-lactalbumin, and β-casein, in mitigating the UV-induced degradation of folic acid (FA). Their hypothesis suggests that the interaction between proteins and FA forms complexes that diminish the exposure of FA to oxidative agents responsible for its degradation, thereby enhancing its stability. Additionally, samples containing CaMTFA exhibited comparable degradation behavior to the corresponding samples containing MTFA under both dark and light conditions. This indicates that due to their similar chemical state in aqueous solution, the solubilized MTFA and CaMTFA show a similar chemical stability. Calcium is an vital micronutrient crucial for all individuals, particularly children, pregnant women, and the elderly. Inadequate calcium intake can lead to nutritional disorders such as rickets, cramps, and osteomalacia/osteoporosis [60]. CaMTFA has become increasingly popular as a source of folate in fortified foods and nutritional supplements. However, the incorporation of Ca^2+^ in food products may trigger the aggregation of caseinates, thereby compromising the stability of the complexes and diminishing their protective efficacy for bioactive small molecules. Understanding the impact of Ca^2+^ on the formation process and interactions of the NaCas-folate complex is crucial for designing and optimizing the formulation of protein-based folate-fortified foods. The findings of the study indicate that the incorporation of a small amount of calcium ion does not change the protein conformation, hence not affecting the interaction of NaCas with MTFA, as well as its degradation derivatives. Paul et al. [41] demonstrated that caseinate aggregation is observable only when the Ca^2+^ concentration reaches 2 mM. Considering the relatively low addition levels of CaMTFA in food products, the risk of protein conformational changes induced by Ca^2+^ from CaMTFA is minimal.

### 3.8. Thermal Stability of Protein by DSC

**Figure 7:**
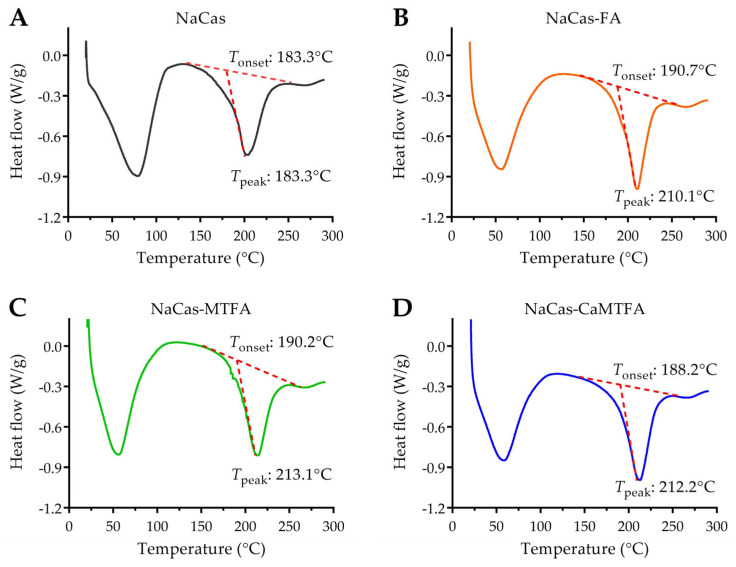
DSC thermograms of sodium caseinate (NaCas) alone (**A**) and NaCas complexes with folic acid (FA), 5-methyltetrahydrofolate (MTFA), and calcium 5-methyltetrahydrofolate (CaMTFA) (**B**–**D**). The onset temperature (*T*_onset_) and peak temperature (*T*_peak_) were marked, which were calculated automatically by TA Universal Analysis software.

DSC represents a valuable thermal analysis tool for assessing protein stability through the examination of its thermal denaturation behavior under controlled heating conditions. The onset temperature (*T*_onset_) and peak temperature (*T*_peak_) determined by DSC are indicative of the initial denaturation temperature and maximum degradation temperature, respectively, providing insights into protein stability [61]. The DSC thermogram of the NaCas sample displayed two distinct broad endothermic peaks, corresponding to water evaporation (25–100 °C) and caseinate degradation (180–250 °C), as illustrated in Figure 7. This result was in agreement with the report by Zhang et al. [62]. The thermal degradation behavior of NaCas was observed to initiate at 183.3 °C (*T*_onset_) and peak at 203.0 °C (*T*_peak_). Upon complex formation with folates, the endothermic peak associated with the degradation of NaCas complexes exhibited a slight shift towards higher temperatures, with *T*_onset_ and *T*_peak_ values of approximately 190 °C and 210 °C, respectively. Previous research has indicated that certain small molecules can stabilize protein conformation through non-covalent interactions, thereby improving protein stability [63]. The findings of this study suggest that the interaction with folate molecules contributes to the enhanced thermal stability of caseinate.

## 4. Conclusions

This study examined the interaction between two commercial folate molecules (FA and MTFA) and NaCas, as well as their impact on the chemical stability of these folates in liquid food products. Fluorescence analysis revealed that both FA and MTFA led to typical static quenching of NaCas. Thermodynamic analysis and molecular docking further demonstrated that NaCas spontaneously bound to FA and MTFA (ΔG < 0), with the binding of FA primarily driven by entropy (ΔH > 0, ΔS > 0) and the binding of MTFA primarily driven by enthalpy (ΔH < 0, ΔS < 0). In the formation of the FA- and MTFA-NaCas complexes, hydrophobic and hydrogen bond forces are identified as key contributors. Experimental findings on storage stability indicate that caseinate-FA/MTFA complexes exhibit notable resistance to rapid degradation. Furthermore, the impact of 25 μM Ca^2+^ on the properties of the NaCas-MTFA complex was explored. The results suggest that the presence of low concentrations of Ca^2+^ minimally influences the binding mechanism of the NaCas-MTFA complex and the degradation behavior of MTFA due to the negligible effect of low Ca^2+^ concentrations on the caseinate conformation. This study represents the inaugural investigation of the interactions and characteristics of caseinate and MTFA complexes in the presence or absence of Ca^2+^. The findings demonstrates the promising potential of caseinates as vehicles for encapsulating folates and offer a theoretical framework for developing formulation approaches for commercially produced folate-fortified beverages.

## Figures and Tables

**Table 1:** Quenching constants (*K*_q_), binding constants (*K*_a_), binding site numbers (n) and thermodynamic parameters of caseinate-folate systems with at three different temperatures. The data were expressed as the mean ± standard deviation of three replicates. All fitting data are shown in Table S2.

System	T (K)	*K*_q_ (×10^12^ L·mol^−1^·s^−1^)	*K*_a_ (×10^4^ M^−1^)	n	ΔG (kJ·mol^−1^)	ΔH (kJ·mol^−1^)	ΔS (J·mol^−1^ K^−1^)
FA	298.2	4.87 ± 0.03	7.84 ± 0.09	1.02 ± 0.01	−27.92 ± 0.03	21.65 ± 0.49	166.30 ± 1.62
	303.2	4.66 ± 0.01	9.00 ± 0.13	1.04 ± 0.02	−28.74 ± 0.03		
	308.2	4.97 ± 0.02	10.41 ± 0.17	1.05 ± 0.01	−29.58 ± 0.04		
MTFA	298.2	5.09 ± 0.02	13.70 ± 0.23	1.07 ± 0.01	−29.30 ± 0.04	−45.62 ± 1.30	−54.79 ± 4.29
	303.2	4.25 ± 0.02	9.96 ± 0.16	1.06 ± 0.01	−28.99 ± 0.04		
	308.2	3.90 ± 0.02	7.53 ± 0.05	1.04 ± 0.01	−28.76 ± 0.02		
CaMTFA	298.2	5.27 ± 0.02	13.68 ± 0.02	1.06 ± 0.01	−29.30 ± 0.01	−46.09 ± 1.96	−56.28 ± 6.47
	303.2	4.24 ± 0.03	10.29 ± 0.05	1.06 ± 0.01	−29.07 ± 0.01		
	308.2	3.76 ± 0.02	7.48 ± 0.06	1.04 ± 0.01	−28.73 ± 0.02		

**Table 2:** Protein secondary structure contents of NaCas-folates systems.

Formulation	α-Helix (%)	β-Sheet (%)	β-Turn (%)	Random Coil (%)
NaCas	19.60 ± 0.14	25.71 ± 1.04	36.06% ± 1.27	18.63 ± 0.31
NaCas-FA	19.52 ± 0.10	26.36 ± 0.01	35.45% ± 0.26	18.67 ± 0.23
NaCas-MTFA	19.80 ± 0.27	25.59 ± 1.15	35.93% ± 1.15	18.68 ± 0.27
NaCas-CaMTFA	19.42 ± 0.07	26.09 ± 1.29	35.93% ± 1.34	18.56 ± 0.12

**Table 3:** The changes in the particle size and ζ-potential of NaCas-folates complexes before and after storage.

Condition	NaCas		NaCas + FA	
	Particle Size (nm)	ζ-Potential (mV)	Particle Size (nm)	ζ-Potential (mV)
0 days	168.6 ± 7.0	−16.4 ± 1.2	165.6 ± 6.1	−17.4 ± 0.8
7 days in light	168.0 ± 0.4	−17.8 ± 0.8	137.4 ± 5.4	−21.7 ± 1.6
49 days in dark	167.7 ± 3.8	−16.7 ± 1.7	144.0 ± 4.4	−19.1 ± 1.2
Condition	NaCas + MTFA		NaCas + CaMTFA	
	Particle size (nm)	ζ-potential (mV)	Particle size (nm)	ζ-potential (mV)
0 days	172.6 ± 5.1	−16.3 ± 0.9	174.6 ± 0.3	−16.3 ± 0.2
3 days in light	139.2 ± 2.3	−26.2 ± 0.5	137.8 ± 2.0	−26.4 ± 1.4
7 days in dark	142.8 ± 6.2	−23.6 ± 0.3	144.9 ± 1.6	−23.1 ± 1.0

## Data Availability

All data are available from the corresponding author when required.

## Supplementary Materials

The following supporting information can be downloaded at: https://www.mdpi.com/article/10.3390/foods13172756/s1, Figure S1. Van’t Hoff plots for the binding interaction of NaCas with folic acid (FA) and 5-methyltetrahydrofolate (MTFA), as well as MTFA under 25 µM divalent calcium ion (Ca^2+^); Figure S2. FT-IR spectra of of NaCas alone and NaCas-folate complexes with folic acid (FA), 5-methyltetrahydrofolate (MTFA), and calcium 5-methyltetrahydrofolate (CaMTFA); Figure S3. Ramachandran plot of predicted αs1-casein and β-casein model; Table S1. The calculated lowest binding free energies of molecular docking conformations between FA/MTFA and αs1-casein/β-casein; Table S2. The fitting results of fluorescence data for caseinate-folate systems based on the Stern-Volmer equation and Lineweaver-Burk equations; Table S3. The linear regression curve of the fluorescence intensity versus protein concentration for calculating the protein Surface hydrophobicity (S_0_) value.
