# Supplementary material for: Non-Covalent Interaction of Folic Acid and 5-Methyltetrahydrofolate with Caseinates Improves the Folates Stability Studied by Multi-Spectroscopic Analysis and Molecular Docking
Source: Foods. 2024 Aug 29;13(17):2756. doi: 10.3390/foods13172756 (PMC11394995; doi:10.3390/foods13172756)
Supplement: Supplementary file 1 [file foods-13-02756-s001.zip › foods-3122127-supplementary.pdf]

Supplementary Materials:

Non-covalent Interaction of Folic Acid and 5-Methyltetrahydrofolate with Caseinates Improves the Folate Stability Studied by Multi-spectroscopic Analysis and Molecular Docking

Linlin He [1], Yuqian Yan [1], Gang Zhang [1], Yanna Zhao [1], Fa Zhao [2], Zhuang Ding [1,*], and Zhengping Wang [a]

[1] Institute of Biopharmaceutical Research, Liaocheng University, Liaocheng 252059, China

[2] Shandong Institute for Food and Drug Control, Jinan 250101, China

* Correspondence: dingzhuang@lcu.edu.cn (Z. D.); Tel./Fax: +86-635-8239136 (Z. D.)

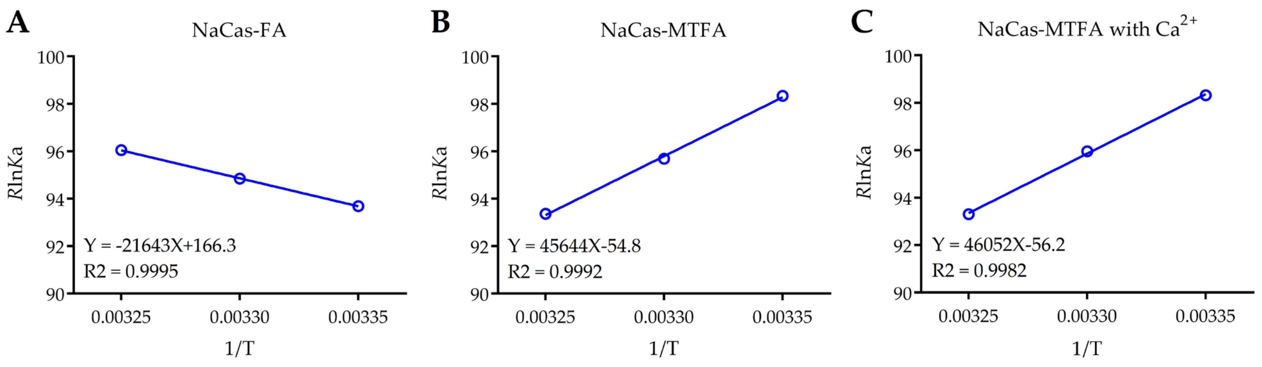

Figure S1. Van't Hoff plots for the binding interaction of NaCas with folic acid (FA) and 5-methyltetrahydrofolate (MTFA), as well as MTFA under 25 μM divalent calcium ion (Ca^{2+}).

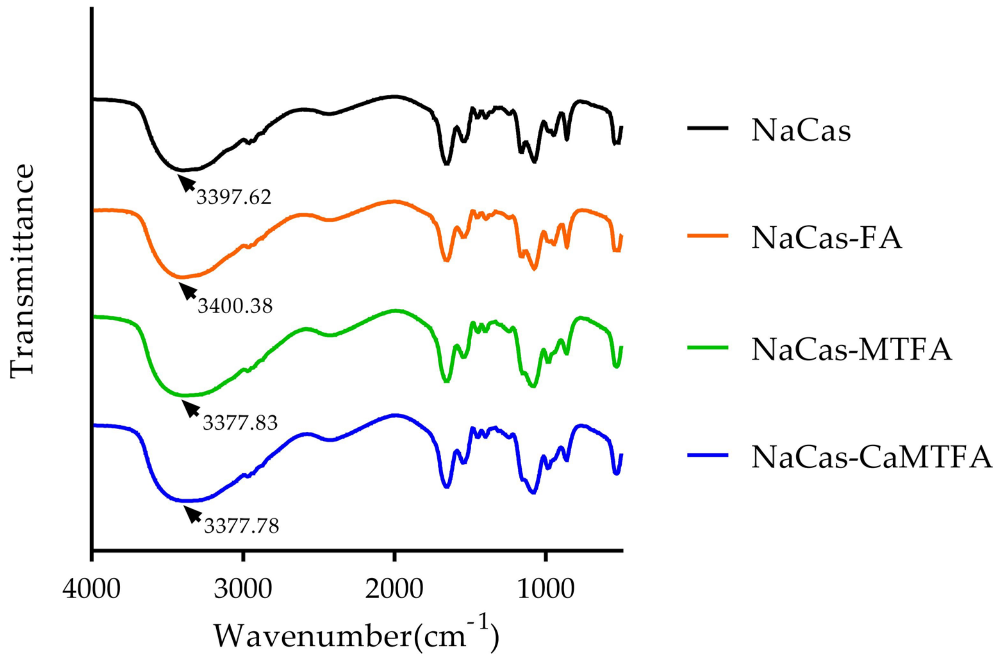

Figure S2. FT-IR spectra of NaCas alone and NaCas-folate complexes with folic acid (FA), 5-methyltetrahydrofolate (MTFA), and calcium 5-methyltetrahydrofolate (CaMTFA).

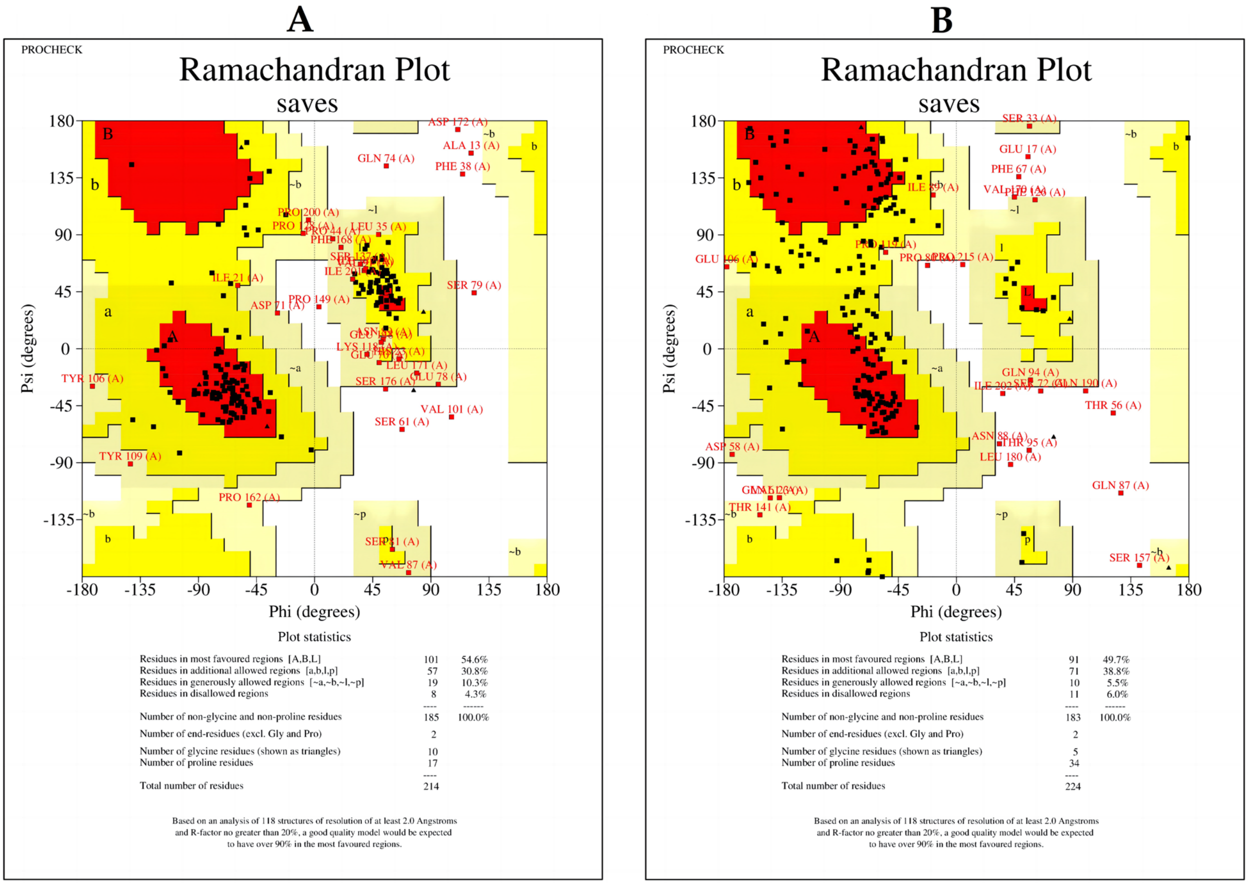

Figure S3. Ramachandran plot of predicted α_{s1}-casein and β-casein models.

Table S1 The calculated lowest binding free energies of molecular docking conformations between FA/MTFA and αs1-casein/β-casein.

System	αs1-casein-FA	β-casein-MTFA	αs1-casein-FA	β-casein-MTFA
Order	Calculated binding free energy (kcal/mol)			
1	-28.9	-30.1	-35.6	-37.2
2	-28.9	-30.1	-35.5	-37.2
3	-27.7	-30.0	-35.3	-37.2
4	-27.7	-29.5	-35.1	-37.2
5	-27.7	-29.5	-35.1	-37.1
6	-26.4	-29.5	-35.1	-36.9
7	-26.4	-29.3	-35.1	-36.9
8	-26.4	-29.3	-34.6	-36.9
9	-26.3	-29.3	-34.6	-36.9

Table S2 The fitting results of fluorescence data for caseinate-folate systems based on the Stern-Volmer equation and Lineweaver-Burk equations.

System	T (K)	Stern-Volmer equation [a]	R^2	Lineweaver-Burk equation [b]	R^2
FA	298.2	Y=48462X+0.9628	0.9949	Y=1.0203X+4.8859	0.9997
		Y=48751X+0.9612	0.9942	Y=1.0217X+4.8902	0.9995
		Y=48962X+0.9607	0.9991	Y=1.0227X+4.9008	0.9994
	303.2	Y=46576X+0.9684	0.9972	Y=1.0371X+4.9543	0.9995
		Y=46605X+0.9674	0.9969	Y=1.0358X+4.9475	0.9997
		Y=46685X+0.9671	0.9968	Y=1.0385X+4.9610	0.9995
	308.2	Y=49595X+0.9601	0.9959	Y=1.0428X+5.0010	0.9999
		Y=49700X+0.9598	0.9958	Y=1.0441X+5.0173	0.9999
		Y=49961X+0.9589	0.9996	Y=1.0452X+5.0268	0.9999
MTFA	298.2	Y=50648X+0.9498	0.9923	Y=1.0702X+5.1494	0.9998
		Y=50862X+0.9494	0.9952	Y=1.0672X+5.1367	0.9997
		Y=51099X+0.9490	0.9970	Y=1.0655X+5.1303	0.9997
	303.2	Y=42323X+0.9651	0.9933	Y=1.0587X+5.0049	0.9997
		Y=42540X+0.9653	0.9951	Y=1.0567X+4.9983	0.9998
		Y=42673X+0.9658	0.9953	Y=1.0547X+4.9907	0.9998
	308.2	Y=39014X+0.9752	0.9972	Y=1.0410X+4.8735	0.9998
		Y=39392X+0.9739	0.9965	Y=1.0410X+4.8770	0.9996
		Y=39410X+0.9730	0.9961	Y=1.0416X+4.8792	0.9996
CaMTFA	298.2	Y=52465X+0.9486	0.9992	Y=1.0637X+5.1360	0.9995
		Y=52702X+0.9476	0.9963	Y=1.0634X+5.1362	0.9996
		Y=52884X+0.9448	0.9987	Y=1.0640X+5.1379	0.9992
	303.2	Y=42737X+0.9669	0.9963	Y=1.0590X+5.0146	0.9999
		Y=42375X+0.9678	0.9963	Y=1.0593X+5.0124	0.9999
		Y=42206X+0.9679	0.9963	Y=1.0592X+5.0098	0.9999
	308.2	Y=37285X+0.9782	0.9980	Y=1.0424X+4.8697	0.9997
		Y=37557X+0.9782	0.9981	Y=1.0424X+4.8738	0.9996
		Y=37783X+0.9785	0.9981	Y=1.0436X+4.8772	0.9995

[a] In the Stern-Volmer equation, Y represents the ratio of the corrected fluorescence intensities of protein and protein-ligand complexes (F_0/F); X represents the molar concentration of folate ([Q]); The slope of the equation represents the Stern-Volmer quenching constant K_{sv}.

[b] In the Lineweaver-Burk equation, Y represents the base-10 logarithm of $F_0/F - 1$; X represents the base-10 logarithm of the molar concentration of folate ([Q]); The intercept of the equation represents the base-10 logarithm of the binding constant (Ka); The slope of the equation represents the number of binding sites (n) in a bimolecular complex.

Table S3 The linear regression curve of the fluorescence intensity versus protein concentration for calculating the protein Surface hydrophobicity (S_0) value.

System	Fitting equation	Slope of the curve	R^2	S_0
NaCas	y = 0.0334x + 0.0092	0.0334	0.9995	0.0332 ± 0.0003
	y = 0.0333x + 0.0113	0.0333	0.9996	
	y = 0.0329x + 0.0086	0.0329	0.9996	
NaCas-FA	y = 0.0259x + 0.0065	0.0259	0.9972	0.0266 ± 0.0006
	y = 0.0269x + 0.0007	0.0269	0.9997	
	y = 0.0271x + 0.0014	0.0271	0.9996	
NaCas-MTFA	y = 0.0337x + 0.0142	0.0337	0.9994	0.0338 ± 0.0001
	y = 0.0338x + 0.0136	0.0338	0.9990	
	y = 0.0339x + 0.0125	0.0339	0.9993	
NaCas-CaMTFA	y = 0.0342x + 0.0152	0.0342	0.9993	0.0340 ± 0.0003
	y = 0.0337x + 0.0137	0.0337	0.9983	
	y = 0.0340x + 0.0179	0.0340	0.9991	
